# Unraveling the Complex Interactions: Machine Learning Approaches to Predict Bacterial Survival against ZnO and Lanthanum-Doped ZnO Nanoparticles

**DOI:** 10.3390/antibiotics13030220

**Published:** 2024-02-27

**Authors:** Diego E. Navarro-López, Yocanxóchitl Perfecto-Avalos, Araceli Zavala, Marco A. de Luna, Araceli Sanchez-Martinez, Oscar Ceballos-Sanchez, Naveen Tiwari, Edgar R. López-Mena, Gildardo Sanchez-Ante

**Affiliations:** 1 Tecnologico de Monterrey, Escuela de Ingeniería y Ciencias, Av. Gral Ramón Corona No. 2514, Colonia Nuevo México, Zapopan 45121, Jalisco, Mexico; diegonl@tec.mx (D.E.N.-L.); yocan@tec.mx (Y.P.-A.); araceli.zavala@tec.mx (A.Z.); mdeluna@tec.mx (M.A.d.L.); edgarl@tec.mx (E.R.L.-M.); 2 Departamento de Ingenieria de Proyectos, Centro Universitario de Ciencias Exactas e Ingenierias (CUCEI), Universidad de Guadalajara, Av. José Guadalupe Zuno # 48, Industrial Los Belenes, Zapopan 45157, Jalisco, Mexico; araceli.sanchez46@academicos.udg.mx (A.S.-M.); oscar.ceballos@academicos.udg.mx (O.C.-S.); 3 Center for Research in Biological Chemistry and Molecular Materials (CiQUS), University of Santiago de Compostela, Rúa Jenaro de La Fuente S/N, 15782 Santiago de Compostela, Spain

**Keywords:** antibacterial, nanoparticles, lanthanum, machine learning, ZnO

## Abstract

The rise in antibiotic-resistant bacteria is a global health challenge. Due to their unique properties, metal oxide nanoparticles show promise in addressing this issue. However, optimizing these properties requires a deep understanding of complex interactions. This study incorporated data-driven machine learning to predict bacterial survival against lanthanum-doped ZnO nanoparticles. The effect of incorporation of lanthanum ions on ZnO was analyzed. Even with high lanthanum concentration, no significant variations in structural, morphological, and optical properties were observed. The antibacterial activity of La-doped ZnO nanoparticles against Gram-positive and Gram-negative bacteria was qualitatively and quantitatively evaluated. Nanoparticles induce 60%, 95%, and 55% bacterial death against Escherichia coli, Pseudomonas aeruginosa, and Staphylococcus aureus, respectively. Algorithms such as Multilayer Perceptron, K-Nearest Neighbors, Gradient Boosting, and Extremely Random Trees were used to predict the bacterial survival percentage. Extremely Random Trees performed the best among these models with 95.08% accuracy. A feature relevance analysis extracted the most significant attributes to predict the bacterial survival percentage. Lanthanum content and particle size were irrelevant, despite what can be assumed. This approach offers a promising avenue for developing effective and tailored strategies to reduce the time and cost of developing antimicrobial nanoparticles.

## 1. Introduction

Multidrug-resistant bacteria pose significant risks to human health [1]. Antibiotics are commonly administered to combat bacterial infections through various mechanisms, such as targeting prokaryotic cell wall synthesis, protein translation, and DNA replication machinery [2]. However, some bacteria have developed resistance or tolerance mechanisms, including expressing enzymes that degrade antibiotics [3], adapting their cellular components like cell walls and ribosomes [4], and expressing efflux pumps that protect against numerous antibiotics [5]. Some clinically relevant bacteria that have adopted these metabolic properties are the Gram-negative *Escherichia coli*, *Pseudomonas aeruginosa*, and the Gram-positive *Staphylococcus aureus* [6]. These microorganisms cause gastroenteritis, skin infections, and various systemic infections [7,8].

In addition to their size, metal oxide nanoparticles (NPs) possess physicochemical properties like charge, a high surface-to-mass ratio, and high reactivity that can enhance antimicrobial activity [9]. As a result, various NPs composed of gold, silver, and metal oxides have been developed [10,11,12].

Zinc oxide (ZnO) is a semiconductor with a hexagonal wurtzite structure and a wide direct band gap of 3.4 eV, along with an excitation binding energy of 60 meV. Due to its unique properties, ZnO has been utilized in electronic devices such as thin-film transistors, photovoltaics, gas sensors, and biomedical sensors. Additionally, ZnO exhibits biotechnological properties, including antibacterial and antineoplastic activity. ZnO NPs may exert antimicrobial activity by changing cell polarity, producing oxygen-reactive species (ROS) and free radicals, and activating signal transduction pathways [12,13,14]. The antibacterial activity of doped ZnO NPs is associated with variation in the optical band gap and particle size. ZnO has been approved by the Federal Drug Administration (FDA, Rockville, MD, USA) for use as an alternative therapy. These properties can be further enhanced by manipulating its morphology, which is influenced by the synthesis route. A wide variety of synthesis methods have been developed for obtaining ZnO, including green chemistry, combustion-assisted methods, microwave synthesis, and hydrothermal methods, among others [13,14,15].

Lanthanum, a rare earth element, has been used as a doping element or oxide in biomedical applications such as drug delivery, anticancer, and magnetic resonance imaging (MRI) [16,17,18,19]. In all these applications, there is an improvement in the desired characteristics after lanthanum doping. In recent reports, lanthanum-doped ZnO nanoparticles have been primarily prepared using solution-based methods such as chemical precipitation, sol-gel routes, and green synthesis. The resulting morphologies typically include nanoparticles and nanorods. Lanthanum nitrate is commonly used as the precursor due to its water solubility [20,21,22].

Machine learning is a powerful tool for modeling and understanding complex phenomena in various fields, including science, engineering, finance, and healthcare [23]. Machine learning is a form of artificial intelligence that trains models on large datasets to accurately predict future outcomes when presented with new input [24]. The modeling process typically begins by collecting and cleaning data that may originate from sensors, databases, or user interactions. This data is then fed into a machine learning algorithm to identify patterns and relationships between variables, which can be used to predict future events or better understand underlying phenomena [25].

Machine learning models adopt many forms, ranging from simple linear regression models to deep learning neural networks [26]. Each model is designed to capture different aspects of the data, and can be adapted to specific use cases [27]. In this work, eight models were built: Multi-Layer Perceptron (MLP), K-Neighbors (KNN), Gradient Boosting (XGB), Extremely Random Trees (ERT), Random Forest (RF), Decision Trees (DT), Linear Regression (LR), and Support Vector Regressor (SVR). Multi-Layer Perceptron (MLP) is a type of feedforward artificial neural network. It consists of multiple layers of nodes (neurons), each connected to the next layer. The input layer receives the initial data, which passes through hidden layers where computation occurs and finally produces an output. MLPs can learn complex patterns in data and are commonly used for classification and regression tasks. They utilize activation functions to introduce non-linearity, allowing them to approximate a wide range of functions. K-Neighbors (KNN) is a simple yet effective supervised learning algorithm for classification and regression tasks. In KNN, the prediction for a new data point is based on the average (for regression) of its k nearest neighbors in the training data. The distance metric, such as Euclidean distance, is typically used to determine the similarity between data points. Gradient Boosting (XGB) is an ensemble learning technique that builds a robust predictive model by combining multiple weak learners sequentially. Each weak learner is trained to correct the errors made by the previous ones.

Gradient Boosting involves minimizing a loss function by iteratively adding decision trees (boosting) to the model. It achieves high predictive accuracy by focusing on areas where previous models perform poorly. Extremely Random Trees (ERT) is an ensemble learning method like Random Forest (RF). However, instead of selecting the best split among a subset of features at each node in a decision tree, ERT randomly selects splits for each feature. This introduces more randomness, which can reduce overfitting and improve generalization performance. Random Forest (RF) is another ensemble learning technique based on decision trees. It builds multiple decision trees during training and outputs the mode (for classification) or the mean prediction (for regression) of the individual trees. RF is robust to overfitting and noise, making it popular for various classification and regression tasks. Decision Trees (DT) are non-parametric supervised learning algorithms for classification and regression tasks. They recursively split the data into subsets based on the values of the features, with each split maximizing the information gain or minimizing impurity. Decision trees are easy to interpret and visualize, making them helpful in understanding the decision-making process in a model. Linear Regression (LR) is a linear approach to modeling the relationship between a dependent variable and one or more independent variables. It assumes a linear relationship between the input variables and the output. LR estimates the coefficients of the linear equation, representing the best-fit line or hyperplane that minimizes the sum of squared differences between the observed and predicted values. Support Vector Regressor (SVR) is a type of support vector machine (SVM) used for regression tasks. SVR works by finding the hyperplane that maximizes the margin between predicted and actual values. It uses a kernel function to map the input variables into a higher-dimensional space where a linear relationship can be found. SVR is effective in handling high-dimensional data, and is robust to outliers. All eight models described can be considered classic machine learning algorithms commonly applied to solve regression problems [28,29,30]. Advantageously, machine learning can handle extensive and complex datasets that would be difficult or impossible to analyze manually [31]. This study fed analytical data into various machine-learning algorithms to model the antibacterial behavior of ZnO doped with lanthanum. To do so, parameters such as the type of bacteria, the dosage of doped NPs applied, treatment time, and cell parameters were considered. The inhibitory effect of the material was represented by the absorbance read from the culture.

**Figure 1:**
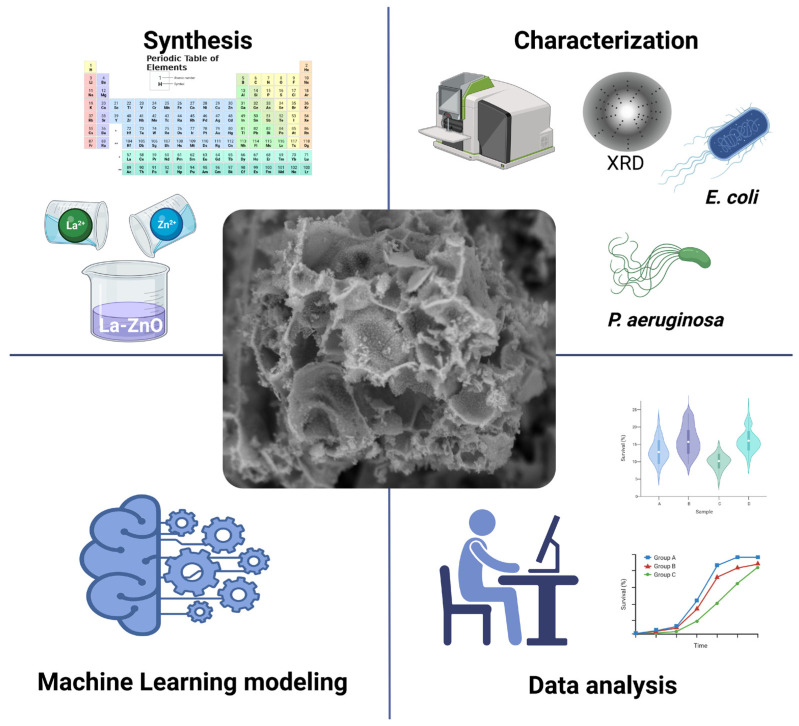
A workflow of the application of machine learning in modeling antibacterial properties of Ln-doped ZnO nanoparticles; XRD—X-ray Powder Diffraction.

Here, we systematically studied the effect of incorporating lanthanum at different concentrations into ZnO prepared via the solution-polymerized method, focusing on their structural, optical, and morphological properties, as well as their relationship with antibacterial activity. Material characterization data were collected to identify the factors contributing most to the antibacterial activity of the doped nanoparticles compared to undoped ZnO. Antimicrobial activity was assessed using the zone of inhibition (ZOI) and bacterial growth curves. Finally, experimental data on bacterial growth kinetics were analyzed using machine learning algorithms to plan future experiments (Figure 1). The best models were optimized using randomized techniques and cross-validation to reduce the likelihood of overfitting. The combination of materials data and antibacterial activity offers new insights into the effectiveness of doping ZnO as an antimicrobial agent.

## 2. Results and Discussion

### 2.1. Nanoparticle Characterization

**Figure 2:**
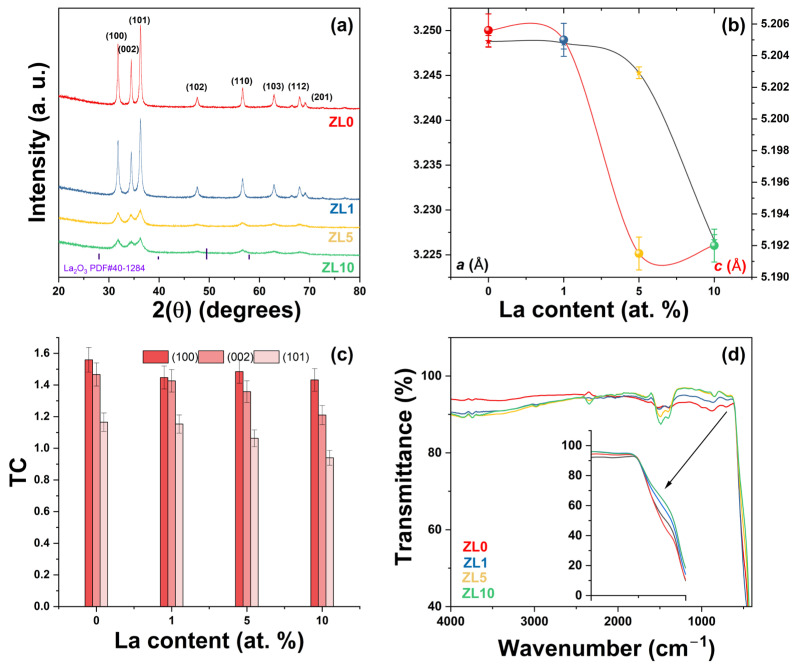
(**a**) X-ray Powder Diffraction (XRD) patterns of ZnO with varying La (Lanthanum) concentrations; the wurtzite ZnO’s lattice planes (hkl) are indicated. (**b**) *a* and *c* lattice parameters calculated from peak positions. (**c**) Texture coefficient (TC) of different planes and (**d**) Fourier Transform Infrared Spectroscopy (FTIR) spectra of undoped and doped ZnO nanoparticles.

Undoped and La-doped ZnO NPs were prepared by solution polymerization. The X-ray diffraction (XRD) patterns of the Zn_1−X_La_X_O (x = 0, 1, 5 y 10 at.%) NPs are shown in Figure 2a. All XRD patterns matched the standard JCPDS # 36-1451, corresponding to the ZnO hexagonal wurtzite structure. All samples exhibited the (100), (002), (101), (102), (110), (103), (200), (112), and (201) diffraction peaks, and no secondary phases related to La^3+^ were observed. As confirmation, the patterns of the doped ZnO NPs were compared with those of the standard La_2_O_3_ (JCPDS # 40-1284), and not one peak of this phase was observed. Crystallinity decreased as La^3+^ content increased (Figure 2a). The samples were labeled as ZL0 for the undoped ZnO, ZL1, ZL5, and ZL10 for respective concentrations (1, 5, 10 at.%).

Table 1 summarizes the structural parameters that characterize the undoped and doped ZnO NPs. Slight variations in 2(θ) positions were observed, which can be attributed to La^3+^ incorporation into the ZnO structure and differences in the radii between La^3+^ (1.16 Å) and Zn^2+^ (0.74 Å) [32]. The *a* and *c* lattice parameters were then calculated [33] (Figure 2b). As La^3+^ content increased, the *a* and *c* lattice parameters decreased due to two phenomena: First, a compressive hydrostatic pressure arises when doping ions are introduced in a ZnO host. Second, when doping ions (La^3+^) localize in the nonequilibrium position, these ions shift toward an equilibrium position. Similar results have been reported after doping ZnO with rare earth elements [34,35].

Structural parameters were modified as La^3+^ content increased (Table 1). The *c/a* ratio or packing factor for an ideal stoichiometric structure is 1.6333. An *R* (degree of distortion) value greater or less than unity indicates the presence of zinc and oxygen vacancies [36]. The average crystallite size (*D*) was calculated from the Scherrer equation [37]. The *D* value decreased due to a reduced growth rate [38]. The texture coefficient (TC) was calculated from the XRD analysis to evaluate the effect of La^3+^ incorporation on preferred orientation growth using the Harrys texture equation [39]. According to the literature, the preferred orientation growth is determined by the interaction between solvents and crystal planes of the NPs [40]. Strong interactions between solvent-crystal surfaces reduce growth in a particular crystal plane. No significant changes were observed in the TC value for the (100) plane (Figure 2c). More significant variation was observed for the (002) and (101) planes. Upon incorporating various ions from the original lattice (ZnO), the nucleation type changes from homogenous to heterogeneous due to forming a new nucleation center [41].

The presence of functional groups on the surface of the NPs was confirmed by Fourier-transform infrared spectroscopy (Figure 2d). All NPs exhibited similar spectra; the peak of ~3765 cm^−1^ can be attributed to carbon from measurement residues [42]. The 3700–3584 cm^−1^ absorption peaks correspond to the –OH stretching vibration from water adsorbed on the particle surface. A weak band localized at ~2995 cm^−1^ corresponded to C–H stretching vibration. The band at ~2346 cm^−1^ was due to atmospheric CO_2_. The strong intensity peaks between 1490 and 1400 cm^−1^ were related to C–H bending vibration. A band at ~893 cm^−1^ corresponded to the formation of tetrahedral coordination of Zn. Another band at ~710 cm^−1^ was related to the stretching vibration of ZnO particles. The broad absorption band observed at ~460 cm^−1^ confirmed the successful formation of metal–oxygen (M–O, stretching vibration) from undoped and La-doped NPs. The inset of Figure 2d reveals a slight shift in the M–O band, which can be attributed to lattice distortion and is supported by XRD analysis [43]. The residual organic groups were related to poly(vinyl alcohol) (PVA)/sucrose decomposition during synthesis.

**Figure 3:**
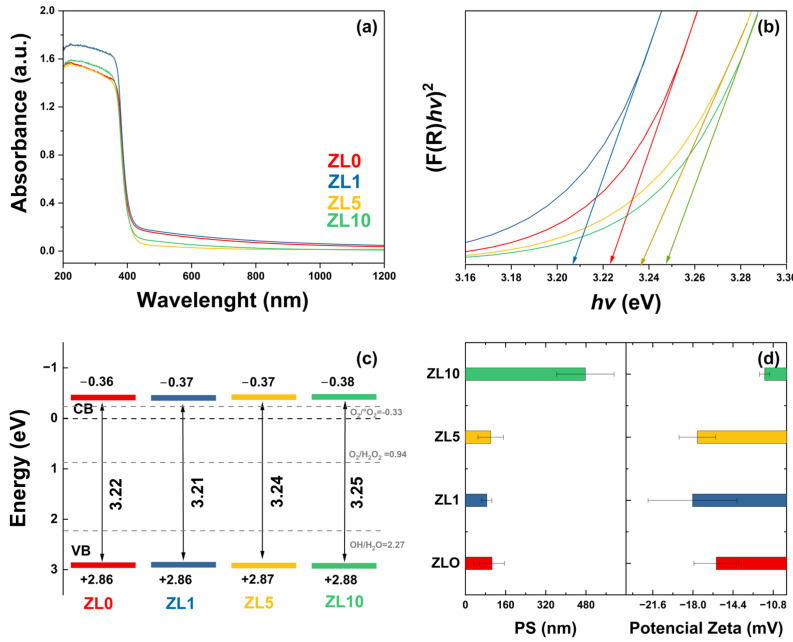
(**a**) UV–Vis spectra. (**b**) Kubelka-Munk function versus energy plots to determine band gap for undoped and doped ZnO nanoparticles. (**c**) Conduction and valence bands energies. (**d**) Particle size (PS) in solution and zeta potential.

The optical properties of undoped and La-doped ZnO NPs were analyzed by UV–vis diffuse reflectance spectroscopy (DRS) (Figure 3a). All samples exhibited an absorption edge of ~365 nm, which can be attributed to the direct band gap of ZnO. This is due to the electron transition from the valence band (VB) to the conduction band (CB) (O2p–Zn3d) [44]. The observed variation in the absorbance spectra is related to defects in features such as crystallite size, grain boundaries, and oxygen vacancies [45]. The band gap was calculated using the absorbance data, the Kubelka–Munk function, and Tauc’s relation (Figure 3b) [46]. The obtained values were 3.22, 3.21, 3.24, and 3.25 eV for ZL0, ZL1, ZL5, and ZL10, respectively. For the ZL1 sample, a red shift was observed. In contrast, the ZL5 and ZL10 samples exhibited a blue shift compared with the ZL0 sample. According to a previous report, red and blue shifts are due to tensile and compressive stress, respectively [47].

The energy of the CB and VB (Figure 3c) were calculated from band gap values [48]; their positions determine the formation of ROS and, therefore, the biological applications of NPs. Here, the CB and VB values included the potential of the superoxide anion and hydroxyl (1.99 and −0.33 eV), which affect the antibacterial properties of the NPs [49]. The size distribution and zeta potential of the NPs in aqueous medium are shown in Figure 3d. According to these values, NPs tended to agglomerate when in suspension. The average size of ZL0 was almost three times higher than the other NPs. The zeta potential of all the samples was less than −30 mV, indicating they are strongly anionic. The ZL10 exhibited lower zeta potential values according to the size distribution results.

**Figure 4:**
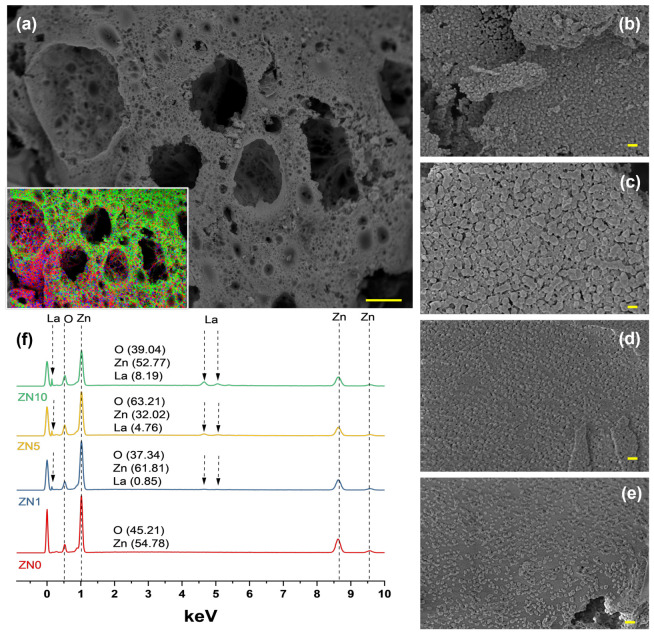
(**a**) Low-magnification Field emission scanning electron microscopy (FESEM) image and energy-dispersive X-ray spectroscopy (EDS) element overlap maps of La-ZnO 5 at.%. (**d**) High-magnification images of (**b**) ZN0, (**c**) ZN1, (**d**) ZN5, and (**e**) ZN10. (**f**) EDS spectrum of all the samples. The scale bar corresponds to 10 mm (**a**) and 100 nm (**b**–**e**).

Field emission scanning electron microscopy was used to evaluate the microstructure of the La-doped ZnO NPs; representative results for 5 at.% La content samples are shown in Figure 4a. PVA was used as a stabilizing agent in this synthesis method to control the growth of NPs, whereas sucrose was used to fuel the reaction. Our previous work described the chemical mechanism in detail [43]. Figure 4a shows that the microstructure consisted primarily of interconnected bubbles and was highly porous, likely due to the presence of organic materials during synthesis. Figure 4a (inset) represents a Zn (red), O (green), and La (blue) elemental map-ping mix. High-magnification images (Figure 4b–e) revealed the average particle sizes of ZL0, ZL1, ZL5, and ZL10 to be 36.4, 35.3, 28.3, and 24.5 ± 5% nm, respectively. The composition of the samples measured using energy-dispersive X-ray spectroscopy (EDS) is shown in Figure 4f.

### 2.2. Potential Role of Particles as Nano-Antibiotics

**Figure 5:**
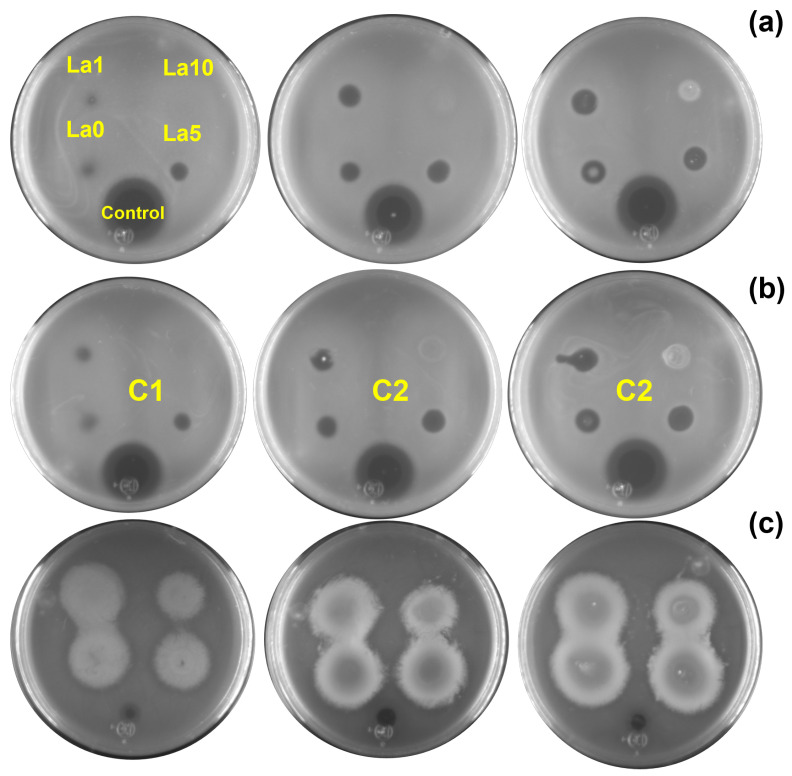
ZOI results obtained for (**a**) *E. coli*, (**b**) *S. aureus*, and (**c**) *P. aeruginosa* exposed to different nanoparticle concentrations (C1 = 5 mg/mL, C2 = 10 mg/mL, C3 = 20 mg/mL).

Two assays were performed to evaluate the antibacterial activity of the Zn_1−X_La_X_O (x = 0, 1, 5, 10 at.%) NPs against (a) *E. coli*, (b) *S. aureus*, and (c) *P. aeruginosa*. First, bacterial inhibition was measured in nutrient agar plates, where a change in the color of the nutrient agar characterized the ZOI. For *E. coli* and *S. aureus*, variations in the agar color increased with NP concentration. However, this effect was unnoticeable for ZL10. Figure 5 shows the ZOI by quantifying the radii in the Petri dish after 24 h of exposure to E. coli, *S. aureus*, and *P. aeruginosa* for each NP and concentration. Inhibition results varied across bacteria. The inhibition of *E. coli* and *S. aureus* was associated with the NP type, not the concentrations, as the ZnO NP generated a greater ZOI. *P. aeruginosa* exhibited similar results following exposure to the La-doped ZnO at 10 at.%.

*P. aeruginosa* employs many virulence mechanisms, equipping the bacteria with advanced properties that enhance the adaptation process inside the host environment. These factors encompass a complex array of secretion (T1SS, T2SS, T3SS, T4SS, T5SS, and T6SS) and communication systems (quorum sensing). Additionally, the bacteria produce proficient lipopolysaccharides and toxins, and employ intricate biochemical mechanisms that allow them to face redox conditions and iron starvation in the host [50].

An interesting effect was observed when *P. aeruginosa* was exposed to NPs. *P. aeruginosa* produced a blue-greenish virulence factor known as pyocyanin (N-methyl, L-hydroxyphenazine), which, together with pyoverdine, highly contributes to the colonization of *P. aeruginosa*. Pyocyanin is a zwitterion with a phenolic group whose redox properties render it highly reactive [51]. Significant levels have been detected in chronic infections associated with sputum sol, ear secretions, and wounds [52]. Pyoverdine is a siderophore that plays an essential role in the uptake of external iron, one of the primary metabolic nutrients that determines the activity of various metalloproteins.

**Figure 6:**
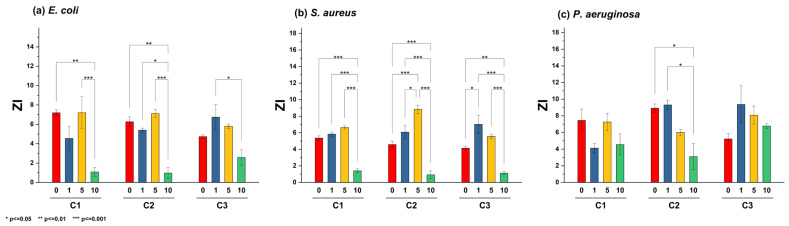
Bar plot and Tukey’s test of zone of inhibition (ZI) in different strains: (**a**) *E. coli*, (**b**) *S. aureus*, and (**c**) *P. aeruginosa*, using different nanoparticle concentrations (C1 = 5 mg/mL, C2 = 10 mg/mL, C3 = 20 mg/mL).

Thus, decreasing the production of both pigments could attenuate the virulence of *P. aeruginosa*, thereby strengthening the host’s natural defense against infection [53]. Many studies have shown the inhibition of this pigment at high NP concentrations, especially when *P. aeruginosa* is exposed to silver NPs [54]. Previous studies have shown that using ZnO nanoparticles promotes a significant decrease in diverse virulence factors exhibited by *P. aeruginosa*, including various clinical isolates. Remarkably, an optimal concentration of 8 mg/mL exhibited high efficiency in diminishing virulence capacity, affecting the production of rhamnolipids, pyoverdine, elastases, and proteases. Furthermore, a downregulation in gene expression associated with the Quorum sensing system was also observed [55]. An assessment employing a concentration profile of ZnO NPs reveals that optimal efficacy in biomass reduction, biofilm formation, and the expression of virulence factors, such as pyocyanin and pyoverdine, is achieved at higher concentrations. The statistical analysis of ZOI data shown in Figure 6 highlights significant differences in these values for the ZL10 sample.

**Figure 7:**
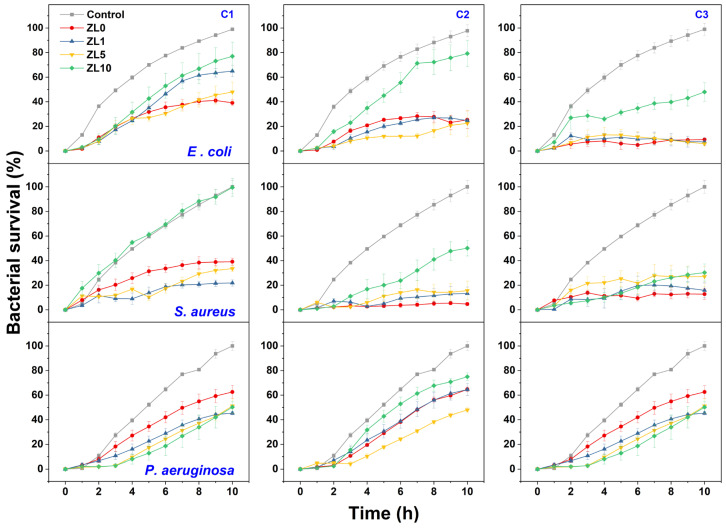
Growth kinetic curves of *E. coli*, *S. aureus*, and *P. aeruginosa* treated with NPs at different concentrations (C1 = 5 mg/mL, C2 = 10 mg/mL, C3 = 20 mg/mL) compared to control culture.

A bacterial growth kinetics assay was used to characterize the antibacterial properties of La-doped ZnO NPs. Figure 7 shows the growth rate of the three bacteria following exposure to NPS at different doping and concentrations (C1 = 5 mg/mL, C2 = 10 mg/mL, C3 = 20 mg/mL). The highest inhibition of bacterial growth compared to the control was measured after 10 h of exposure; this duration was used for further analysis.

At the C1 dose, ZL0 exerted significant antibacterial activity (61%) against *E. coli*, whereas ZL1 treatment proved effective against *S. aureus* with approximately 79% inhibition. *P. aeruginosa* was most inhibited by ZL1 (nearly 55%). The inhibitory effects of ZL0, ZL1, and ZL5 at the C2 concentration were similar (76–78%) against *E. coli.* ZL0 treatment exerted the highest antibacterial activity against *S. aureus*, with nearly 95% inhibition. ZL5 performed best against *P. aeruginosa* (around 51%). At C3, the growth of *E. coli* was the most inhibited when treated with ZL5 (around 95%), whereas *S. aureus* was most inhibited by ZL0 (87%). ZL1 treatment best inhibited (55%) the bacterial density of *P. aeruginosa*. Regardless of dose, ZL10 had lower antibacterial activity against *E. coli* and *S. aureus* strains. Dose-dependent inhibition of bacterial growth was determined for all strains, as bacterial density decreased with increasing doses of NPs.

**Figure 8:**
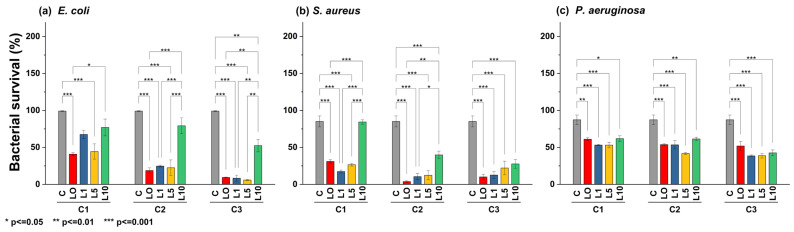
Bar plot and Tukey´s test of bacterial survival percent in different strains: (**a**) *E. coli*, (**b**) *S. aureus*, and (**c**) *P. aeruginosa*, using different nanoparticle concentrations (C1 = 5 mg/mL, C2 = 10 mg/mL, C3 = 20 mg/mL).

According to statistical analysis (Figure 8), *E. coli* was sensitive to ZL0, ZL1, and ZL5 doping at higher concentrations (C3, *p* < 0.001). However, ZL10 was ineffective, allowing the bacteria to reach viability up to 50.50%. *S. aureus* was less sensitive to NPs regardless of doping or concentration. Interestingly, ZL1 inhibited *S. aureus* equally across all concentrations, where bacterial viability reached an average minimum of 15.87%. Despite doping, *P. aeruginosa* was less sensitive to this type of NP, even at high concentrations; all treatments yielded a bacterial viability of 50% or more.

**Figure 9:**
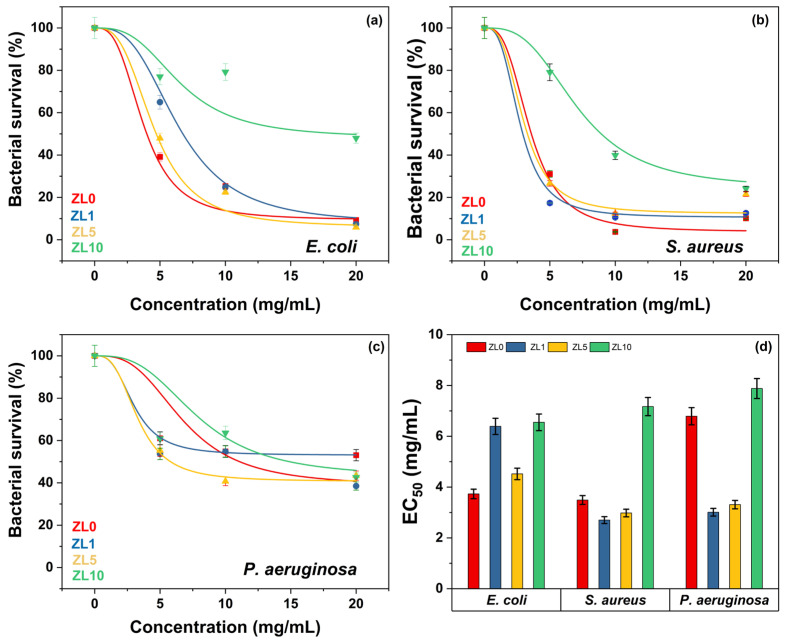
Bacterial viability at various NP concentrations (**a**–**c**). Doped NPs and their respective EC_50_ against bacterial strains (**d**).

The half-maximal effective concentration (EC_50_) is the concentration of a drug or chemical that induces a biological response halfway between the baseline and the maximum effect. In nanomedicine, EC_50_ refers to the potency of NPs that kill cells, change gene expression, or generate other cellular responses. Figure 9a–c shows that, regardless of doping, bacterial survival declined as NP concentration increased. *E. coli* and *S. aureus* were least affected by ZL10 (Figure 9a,b), with EC_50_ values of 6.55 and 7.17 mg/mL, respectively. Even at high concentrations, *P. aeruginosa* was less susceptible to NPs, presenting a bacterial survival above 40% (Figure 9c). Among the three bacteria examined, NPs doped at 10 at.% were the least effective (EC_50_ = 7.20 mg/mL, *p* > 0.05). ZL0 had similar effects on *E. coli* and *S. aureus* with EC_50_ values of 3.73 and 3.49 mg/mL, respectively (Figure 9d). ZL1 elicited a more significant response from *S. aureus* and *P. aeruginosa*, but did not affect *E. coli*. Although *E. coli* and *P. aeruginosa* are Gram-negative bacteria, and *S. aureus* is Gram-positive, the results did not correlate with the Gram-stain, suggesting that the antibacterial activity of NPs is not influenced by membrane composition.

The antibacterial activity of undoped and doped ZnO NPs can be attributed to several potential factors, such as particle size, structural defects (i.e., vacancies), metal ion release, photocatalytic activity, and ROS [56]. In this work, the potential activity of the materials as nano-antibiotics was measured under dark conditions.

Previous studies have attributed the antimicrobial activity of ZnO under dark conditions to superficial defects. Indeed, ROS are produced due to defects responsible for trapped carriers [57] as follows:

(1) $$O_{2}+e^{-}\to {}^{\circ}O_{2}^{-}$$

(2) $${}^{\circ}O_{2}^{-}+H_{2}O\to {}^{\circ}HO_{2}+OH^{-}$$

(3) $${}^{\circ}HO_{2}+{}^{\circ}HO_{2}\to H_{2}O_{2}$$

(4) $$H_{2}O_{2}+{}^{\circ}O_{2}^{-}\to O_{2}+{}^{\circ}OH+OH^{-}$$

In the initial step, an electron from the NP surface reacts with atmospheric oxygen to produce a superoxide radical, which reacts with water to produce hydroperoxyl radicals. Next, these hydroperoxyl radicals recombine to produce hydrogen peroxide. Finally, a reaction between superoxide radicals and hydrogen peroxide enables the formation of hydroxyl ions and hydroxyl radicals. The antibacterial activity of ZnO under dark conditions is related to the °$O_{2}^{-}$ and H_2_O_2_. These ions and radicals destroy the organic groups of the outer cell membrane, killing bacteria.

### 2.3. Machine Learning Modeling

Table 2 summarizes the computational experiments performed to predict bacterial survival. The table reports the time required to train the model in each case and the time to compute the predictions on the test set. The mean absolute error (MAE) value is notably lower for better models. A correlation coefficient *R^2^* is also computed for the training and test sets; if their difference is less than 2%, the model was not overfitted or underfitted. All the models accurately predicted the bacterial survival rate (%).

**Figure 10:**
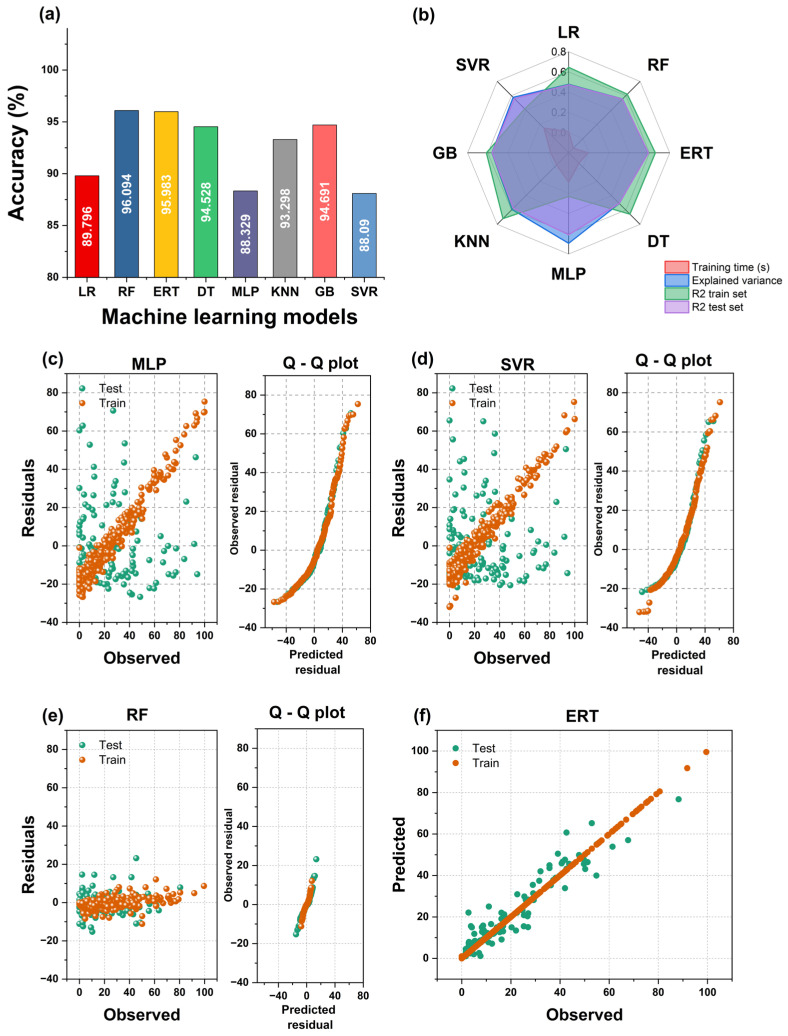
(**a**) Accuracy of the tested models. (**b**) Radar plot depicting the results for the machine learning models used to predict bacterial survival. (**c**–**e**) Residual and quantile–quantile (Q–Q) plots obtained for the machine learning models. (**f**) Observed vs. predicted values of bacterial survival for best model.

Random forest (RF) generated the smallest MAE (about 4%), though the Extremely Random Tree (ERT) model showed very similar results. Remarkably, the R^2^ scores of the decision tree (DT), ERT, and RF models were almost perfect (1.0), although the MAE in those cases hovered around 5%. This may be due to a slight model overfitting, which can be reduced in the workflow’s next step. The multilayer perceptron (MLP) and the support vector regressor (SVR) models performed worst. These results are shown in Figure 10a,b. In addition, Figure 10c–e show the residual and the Q–Q plots of train and test values for representative models. The predicted values of residuals represent normal distribution as the base distribution.

### 2.4. Hyperparameter Optimization

After the training, hyperparameter optimization can be performed to determine the best model. Hyperparameters define the behavior and performance of a machine learning algorithm; they are not learned directly from the data, but instead are assigned before training the model. Examples include learning rate, regularization strength, number of hidden layers, number of nodes per layer, and activation functions. Hyperparameter optimization is crucial in machine learning because it directly impacts the model’s performance. Hyperparameters are settings external to the model and control aspects such as complexity, learning rate, regularization, and more. Finding the right combination of hyperparameters can significantly improve a model’s accuracy, generalization ability, and efficiency. Without proper hyperparameter tuning, a model may suffer from overfitting or underfitting, where it either learns the training data too well but fails to generalize to new data or fails to capture the underlying patterns in the data, respectively. Hyperparameter optimization helps strike a balance between model complexity and generalization by fine-tuning these settings. Moreover, different datasets and problem domains may require different hyperparameter configurations for optimal performance. Therefore, performing hyperparameter optimization ensures that the model is tailored to the specific data characteristics, leading to better results and more robust models. Overall, hyperparameter optimization is essential for maximizing the performance and effectiveness of machine learning models in various tasks and domains.

Hyperparameter optimization techniques include grid search, random search, and Bayesian optimization. This work used random search, which randomly samples hyperparameters from a given search space. Random search is less computationally expensive than grid search, especially in high-dimensional hyperparameter spaces. Grid search evaluates every possible combination of hyperparameters within set ranges, resulting in exponentially more evaluations as the number of hyperparameters increases.

On the other hand, random search randomly samples a subset of hyperparameter combinations, providing an appropriate balance between exploration and exploitation. Second, random search outperforms grid search in discovering optimal hyperparameter configurations. Grid search may overlook critical hyperparameter values that are not in the specified grid. Random search, by definition, explores the hyperparameter space, improving the possibility of identifying promising configurations. Finally, while Bayesian optimization can determine the next set of hyperparameters to assess based on prior findings, it requires additional modeling and computational overhead. Random search, in contrast, is simple to implement and does not rely on any assumptions about the underlying function being optimized, making it a practical choice for hyperparameter optimization in many scenarios.

The process of random search for hyperparameter optimization can be summarized as follows:

1. Define the hyperparameter search space: The first step is to define a range of values for each hyperparameter that will be optimized.
2. Set the number of iterations: Next, determine how many iterations of the random search will be run. This will determine the number of combinations of hyperparameters that will be sampled.
3. Sample hyperparameters: For each iteration, randomly sample a set of hyperparameters from the defined search space.
4. Train the model: Train a model using the sampled hyperparameters.
5. Evaluate the model on a validation set.
6. Store the results: Store the hyperparameters and the corresponding performance metric (e.g., accuracy, F1 score) for each iteration.
7. Select the best hyperparameters: After completing all iterations, select the set of hyperparameters that performed the best on the validation set.

Experimental validation, mainly through techniques like cross-validation, is crucial for assessing the generalization performance of machine learning models on unseen data. While a model may perform well on the training data, its actual test is how effectively it can generalize to new, unseen instances.

Firstly, experimental validation helps detect overfitting, a common pitfall in machine learning where the model learns to memorize the training data rather than capture underlying patterns. By evaluating the model on unseen data, validation techniques reveal whether the model’s performance degrades significantly, indicating overfitting. Secondly, validation provides insights into the model’s ability to generalize across different subsets of the data. Cross-validation, for example, splits the data into multiple subsets, allowing the model to be trained and tested on different combinations of training and validation sets. This process helps in understanding how robust the model is to variations in the data and whether it can make accurate predictions across different scenarios. Furthermore, experimental validation enables comparisons between different models or variations of the same model. By assessing performance metrics on unseen data, researchers and practitioners can objectively evaluate which model performs better and choose the most suitable one for deployment in real-world scenarios.

The results of the random search are shown in Table 3. The accuracy of the RF and ERT models improved by 5.51% and 15.54%, respectively. Tuning the hyperparameters requires cross-validation (in this case, five folds) to reduce variance, select optimal hyperparameters, maximize data utilization, and avoid over-optimistic results. Thus, the results obtained here are more robust than those reported in Table 2, where only one experiment was run. Overall, the best model to predict bacterial survival was an ERT assigned the parameters in the last row of Table 3.

**Figure 11:**
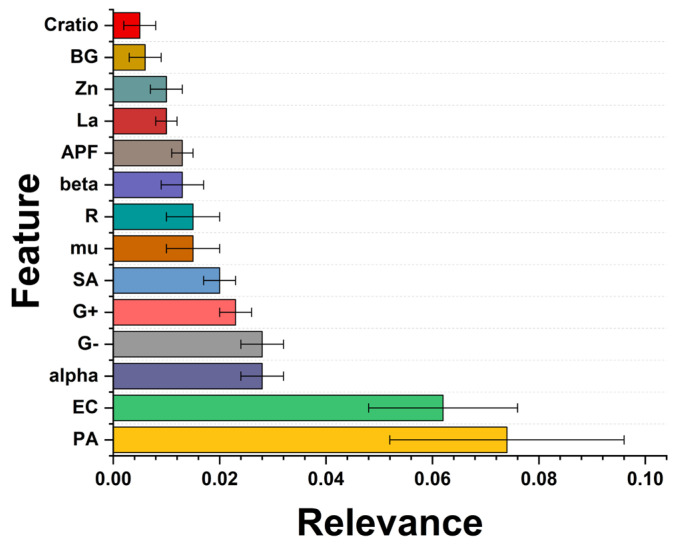
Feature importance results. The bigger the value, the more substantial the contribution of the variable to the model. The values were computed using the permutation importance method.

Finally, a feature importance analysis was performed. Feature importance should be calculated to highlight underlying patterns in the data and identify the most relevant features for making accurate predictions. Here, the permutation importance was performed where the values of a single feature in the test set were randomly changed and subsequent decreases in model performance were measured. The more significant the decrease in performance, the more influential the feature is. Figure 11 reveals the three most influential features in predicting bacterial survival after removing the treatment duration and the dose of the nanomaterial. This is because the antibacterial properties of ZnO have proved that it is dose-dependent [58].

The following relevant feature is the gram type of the bacteria; the most relevant is the Gram-negative type. Regarding the structural parameter of the NPs, the alpha (α-angle) and mu (μ-distance) are related to the Zn–O bond [59], which were modified after lanthanum doping. Nevertheless, the models explored here have not explicitly identified a specific NP characteristic associated with higher antimicrobial activity. Further experimental investigations are needed to optimize the composition of antimicrobial NPs. The other variables are R (lattice distortion), alpha, beta and mu (structural angle), APF (atomic packing factor), La and Zn (lanthanum and zinc content), BG (band gap), and c/a ratio (lattice parameters).

## 3. Methods

### 3.1. Nanoparticles Synthesis

The solution-polymerization method synthesized Zn_1−X_La_X_O (x = 0, 1, 5 y 10 at.%) based nanoparticles [60]. Four solutions were prepared using La^3+^ content (0, 1, 5, 10 at.%). For the preparation, 0.4 g of Polyvinyl alcohol (PVA, Mw: 70,000–1000, Sigma-Aldrich, St. Louis, MO, USA) and 3.0 g of sucrose (C_12_H_22_O_11_, Sigma-Aldrich. ACS reagent, St. Louis, MO, USA) were dissolved in 150 mL of distilled water while stirring at 85 °C for each solution. When the PVA was dissolved, stoichiometric amounts of Zn(NO_3_)_2_6H_2_O (Sigma-Aldrich, St. Louis, MO, USA, 99.9%), as well as La(NO_3_)_36_H_2_O (Sigma-Aldrich, St. Louis, MO, USA, 99.9%), were added, and the solutions were mixed thoroughly while pH was adjusted to 1 with citric acid (C_6_H_8_O_7_H_2_O, Sigma-Aldrich, St. Louis, MO, USA) (Table 4). Furthermore, the solutions were stirred and kept at 85 °C for the evaporation of the remaining water. Subsequently, the obtained foams were dried at 200 °C for 4 h. Finally, the powders were calcined at 450 °C for 4 h in a Thermolyne muffle (Thermo Fischer Scientific, Waltham, MA, USA) under an air atmosphere. All the chemicals were purchased from Sigma-Aldrich. The samples were labeled as ZL0 for the undoped ZnO, ZL1, ZL5, and ZL10 for respective concentrations (1, 5, 10 at.%).

### 3.2. Nanoparticles Characterization Techniques

The crystal structure of the nanoparticles was characterized by XRD (Bruker, Billerica, MA, USA D-8 Advanced diffractometer, rotation activated, Cu anode λ = 1.5406 Å). XRD patterns were obtained from a 20° to 80° (2θ) with a 0.01° step size. Attenuated total reflection Fourier transform infrared (ATR-FTIR) spectroscopy was employed to assess the presence of organic matter in the structure of the nanoparticles. ATR-FTIR spectra were recorded in the 4000–400 cm^−1^ range using an IR Affinity-1S (Shimadzu, Columbia, MD, USA) spectrometer. The morphology of the nanoparticles was investigated using FESEM (TESCAN MIRA3 model, Warrendale, PA, USA). Optical properties were investigated through absorption spectra obtained using a Cary-5000 UV–vis (Agilent Technologies, Santa Clara, CA, USA) spectrometer equipped with a polytetrafluoroethylene (PTFE) integration sphere. A dynamic light scattering instrument (DLS, Microtrac Nanotrac Wave II, Montgomeryville, PA, USA) was used to calculate the average particle size, size distribution, and ζ-potential in water suspensions (1 mg/mL), at pH = 7.

### 3.3. Effect of Nanoparticles on Bacterial Growth

The antibacterial activity of the nanoparticles was evaluated against *Escherichia coli* (ATCC 10536), *Pseudomonas aeruginosa* (ATCC 27853), and *Staphylococcus aureus* (ATCC 33594). A single isolated colony was tacked to inoculate 10 mL of nutrient broth medium (Merck, Millipore, Burlington, MA, USA) and incubated at 37 °C, shaking it at 200 rpm overnight. The Petri dishes consisted of a lower layer of solid LB agar and an upper layer of 10 mL of LB agar 7.5 g/L with 100 μL of the isolated cultured. After solidification, 5 μL of the nanoparticles at different concentrations (5, 10, 20 mg/mL) and 2 μL of 50 μg/mL of Ampicillin (control) were dropped by separated across the surface. The nanoparticles were left to dry. Finally, the Petri dish was incubated at 37 °C for 48 h. The concentrations were labeled as C1, C2, and C3 for 5, 10, and 20 mg/mL, respectively.

### 3.4. Quantitative Determination of the Effect of Nanoparticles on Bacterial Growth

Kinetics data were obtained by monitoring the growth of each bacteria. The optical density (OD600) was measured (Synergy HTX multi-mode reader, Biotek, Agilent Technologies, Inc, Santa Clara, CA, USA) during 8 h, in steps of 1 h. The microplate contained 5 µL of nanoparticle stock solution at C1, C2, and C3 concentrations, glycerol (negative control), and bacteria (positive control), mixed with LB medium to obtain 200 µL per well. The bacterial culture measurements were normalized using controls; samples were analyzed in triplicate.

### 3.5. Statistical Data Analysis

All experiments were done in triplicate, and the results were presented as mean ± standard deviation. The experimental data were analyzed by ANOVA with a post hoc Tukey HSD test with a 95% confidence. Statistical significance was marked with asterisks depending on the *p*-value: * *p* ≤ 0.05, ** *p* ≤ 0.01, *** *p* ≤ 0.001.

### 3.6. Machine Learning Modeling

The goal of neural network modeling is to build a model of the inhibitory effect of La-doped ZnO, using data obtained while characterizing the material and testing it in vitro over three different types of bacteria. The bacterial survival rate (%) is reported as a scalar number, which means the problem is to compute a regression model. The traditional process of creating a Machine Learning regression model involves several key steps (Figure 1) [61]. These steps are as follows:

- Define the problem: The first step is clearly defining the problem you want to solve with the regression model. This includes identifying the input variables (features) and the output variable (target) that you want to predict.
- Collect and preprocess the data: Next, you need to collect and preprocess the data used to train and test the model. This involves cleaning the data, handling missing values, removing outliers, and splitting the data into training and testing sets.
- Choose a regression algorithm: There are several regression algorithms, such as linear regression, polynomial regression, and support vector regression. You need to select the appropriate algorithm based on the problem you are trying to solve and the characteristics of your data.
- Train the model: Once you have chosen the algorithm, you must train the model using the training data. This involves feeding the input data into the algorithm, which will adjust its internal parameters to produce the best possible predictions for the output variable.
- Evaluate the model: After training, you must evaluate its performance using the testing data. This involves measuring how well the model predicts the output variable on data it has not seen before. Standard evaluation metrics include mean squared error (MSE) and R-squared.
- Tune the model: If the model’s performance is unsatisfactory, you can try to improve it by tweaking its parameters or using a different algorithm. This process is called hyperparameter tuning, and involves testing different combinations of parameters to find the best-performing one.
- Deploy the model: Once satisfied with its performance, you can deploy it into production. This involves integrating the model into a software system or application that can use it to make predictions on new data.

In this work, the raw dataset contained 396 rows and 31 columns. The variables in the dataset are the concentration of the doping material, treatment time, dose of the nanomaterial, type of bacteria (*E. coli*, *S. aureus*, *P. aeruginosa*), Gram-staining results, structural, morphological, and optical materials properties. In contrast, the response variable is the bacterial survival rate (%). The dataset was split into two sets: train and test, with a proportion of 70/30% of the total rows, meaning 277 rows for training and 119 for testing. After this separation, the columns with the data for the independent variables were transformed by computing a Min-Max Scaling. After this, all those values were between 0 and 1. It is also important to mention that some variables have categoric values (for instance, the bacteria type or the staining (Gram+ or Gram−)). Those variables were transformed using a One-Hot encoding technique.

With the data prepared, it was possible to compute several models to determine the most promising ones. In this work, eight models were built: Multilayer Perceptron (MLP), K-Neighbors (KNN), Gradient Boosting (XGB), Extremely Random Trees (ERT), Random Forest (RF), Decision Trees (DT), Linear Regression (LR), and Support Vector Regressor (SVR). All the experiments reported in this section were run on an HPZ440 Server (HP Inc, Palo Alto, CA, USA) with a Xeon E51620V3 Processor (Intel, Santa Clara, CA, USA) at 3.5 GHz, 16 GB RAM, 4 Cores, and 8 Processes running Ubuntu 22.04.

In this type of analysis, the first stage usually involves exploring the behavior of various models. Based on each, the subset with the best results is selected, and its hyperparameters are optimized to obtain the best model from a good set of possibilities. The hyperparameters to be optimized depend on each model, and several methods exist to perform this step. In the case reported in this article, an optimization based on a randomized search in the parameter space was used. In any case, the Mean Absolute Error (MAE) and the R2 coefficient were considered suitable metrics to measure the accuracy of the model obtained.

## 4. Conclusions

In summary, antimicrobial La-doped ZnO NPs were fabricated using the solution polymerization method. The incorporation of lanthanum produces lattice parameter contraction and increases the optical band gap. Also, a reduction in the average crystallite size was observed. Three strains of bacteria were more susceptible to NP concentration than material type (lanthanum content %). Interestingly, all the NPs induced pyocyanin production in *P. aeruginosa*, and their antimicrobial activity decreased with increasing lanthanum content. In addition, machine learning algorithms were used to predict the bacterial survival percentage of *E. coli*, *P. aeruginosa*, and *S. aureus* when exposed to different concentrations of La-doped ZnO nanoparticles. The ERT model had the best correlation value (0.96) and the slightest error (3.1%). The hyperparameter optimization analysis showed that NP dose and incubation time contributed the most to the prediction. This study shows that machine learning approaches can inform the rational design of antimicrobial nanomaterials to combat antibiotic-resistant bacteria. With this study, the effectiveness of doping ZnO with lanthanum for antibacterial application was proved. This study can be extended to other nanoparticles where quantitative data on the biological activity are available.

## Figures and Tables

**Table 1:** Structural parameters of prepared nanoparticles. *c* and *a* lattice parameter; *D* the average crystallite size.

Sample	2(θ) (100)	2(θ) (101)	*c/a* Ratio	Unit Cell Vol (Å^3^)	Distortion (*R*)	*D* (nm)
ZL0	31.778	36.277	1.602	47.58	1.019	27
ZL1	31.780	36.280	1.602	45.57	1.019	24
ZL5	31.813	36.267	1.600	47.35	1.020	6
ZL0	32.002	36.405	1.609	46.81	1.015	5

**Table 2:** Metrics for the error and determination coefficient for the predicted values of the complete datasets.

Model	Training Time (s)	Prediction Time (s)	Explained Variance	MAE	R^2^ Train Set	R^2^ Test Set
LR	0.011	0.000	0.4785	10.204	0.6457	0.4722
RF	0.080	0.003	0.9046	3.906	0.9844	0.9022
ERT	0.069	0.003	0.9003	4.017	0.9999	0.9047
DT	0.001	0.000	0.7917	5.472	1.0	0.791
MLP	0.258	0.000	0.3787	11.671	0.2860	0.3619
KNN	0.001	0.081	0.7504	6.702	0.9037	0.7282
GB	0.048	0.001	0.862	5.309	0.9393	0.8595
SVR	0.008	0.004	0.2664	11.91	0.1689	0.2555

**Table 3:** Results of tuning the hyperparameters of the two models that performed the best in the first set of experiments (RF and ERT). The * in the name of the method indicates the optimized version.

Model	Parameters	MAE	Accuracy	R^2^ Score
Random Forest	Default	3.96	58.16	0.90
Random Forest *	{‘n_estimators’: 500, ‘min_samples_split’: 2, ‘min_samples_leaf’: 1, ‘max_features’: ‘sqrt’, ‘max_depth’: 60, ‘bootstrap’: False}	3.98	58.80	0.91
Extremely Randomized Trees	Default	3.98	53.89	0.90
Extremely Randomized Trees *	n_estimators: 522, min_samples_split: 2, min_samples_leaf: 1, max_features: sqrt, max_depth: 20, criterion: log2, bootstrap: False	3.48	62.27	0.95

**Table 4:** Formulation composition.

Sample	PVA (g)	Sucrose (g)	Zn^2+^ Precursor	La^3+^ Precursor
ZL0	0.4	3.0	3.654	0
ZL1	0.4	3.0	3.568	0.065
ZL5	0.4	3.0	3.321	0.254
ZL10	0.4	3.0	3.016	0.487

## Data Availability

The data presented in this study are available on request from the corresponding author.
